# CD38 is a key mediator of NAD^+^ depletion in the brain of ZIKV-infected mice

**DOI:** 10.1016/j.isci.2025.114018

**Published:** 2025-11-12

**Authors:** Georgia N. Saraiva, Bruna G. Sousa, Nicole M.S. Souza, Louise C. Vitorino, Raquel C. da Silva, Thiago S. Bacelar, Matheus O. Atella, Lorena O. Fernandes-Siqueira, Isis Nem de Oliveira Souza, Eduardo Nunes Chini, Juliana Camacho-Pereira, Giselle F. Passos, Andrea T. Da Poian, Julianna D. Zeidler

**Affiliations:** 1 Instituto de Bioquímica Médica Leopoldo de Meis, Universidade Federal do Rio de Janeiro, Rio de Janeiro, Brazil; 2 Instituto de Microbiologia Paulo de Góes, Universidade Federal do Rio de Janeiro, Rio de Janeiro, Brazil; 3 Faculdade de Farmácia, Universidade Federal do Rio de Janeiro, Rio de Janeiro, Brazil; 4 Instituto de Ciências Biomédicas, Universidade Federal do Rio de Janeiro, Rio de Janeiro, Brazil; 5 Department of Anesthesiology and Perioperative Medicine, Mayo Clinic, Jacksonville, FL, USA; 6 Instituto de Biofísica Carlos Chagas Filho, Universidade Federal do Rio de Janeiro, Rio de Janeiro, Brazil

**Keywords:** Neuroscience, Immunology, Virology

## Abstract

Zika virus (ZIKV) infection is a major health concern, particularly during pregnancy, as it can lead to neurodevelopmental delays and congenital brain abnormalities, including microcephaly. Here, we investigated the mechanisms of NAD^+^ depletion in the brains of ZIKV-infected neonatal mice, a model that developmentally corresponds to third-trimester infection in humans. We observed a progressive decline in NAD^+^ levels, which became significant at later stages of infection (18–30 dpi). This decrease did not correlate with viral replication and early *Parp10* or *Parp12* induction, which increased alongside *Nampt* expression, possibly as a compensatory response to NAD^+^ consumption. Instead, NAD^+^ depletion coincided with increased CD38 expression and activity, while CD38 inhibition prevented NAD^+^ loss. Late-stage NAD^+^ depletion was preceded by an induction of inflammatory markers (*Il-6*, *Tnf*, and *Ccl5/Rantes*) and coincided with the infiltration of CD38^+^ immune cells – especially lymphocytes – into the brain, suggesting a link between neuroinflammation and NAD^+^ metabolism dysregulation.

## Highlights

• CD38 is the main driver of NAD^+^ decline during ZIKV infection in the brain
• CD38 activity increases at later stages of infection
• Inflammation precedes NAD^+^ decline and CD38 upregulation in the brain
• This late stage is marked by CD38^+^ immune cell infiltration into the brain

## Graphical abstract

**Figure:**
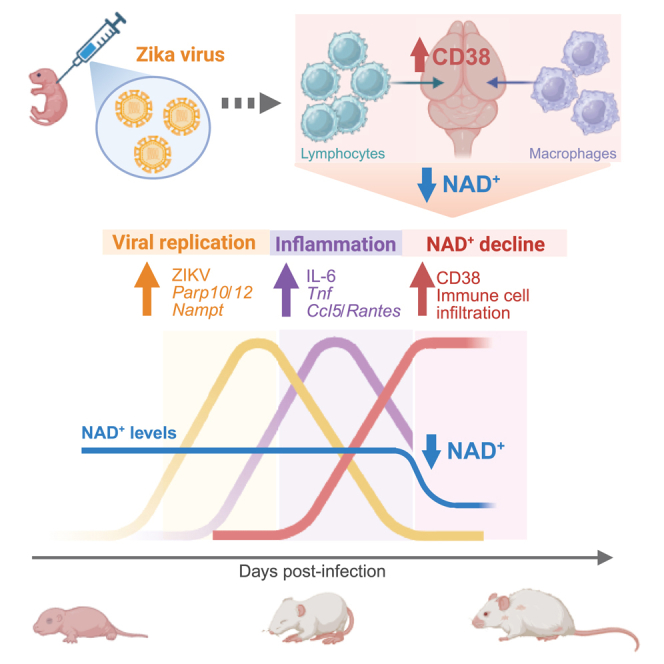

## Introduction

ZIKV is an arbovirus of the *Flaviviridae* family, endemic to tropical and subtropical regions, and primarily transmitted to humans through the bite of *Aedes aegypti* mosquitoes.^1^^,^^2^ In most cases, the infection is asymptomatic or presents mild clinical symptoms – such as fever, headache, and myalgia – in approximately 20% of affected individuals.^3^^,^^4^ In a small percentage of cases, ZIKV infection can lead to severe neurological complications, such as Guillain-Barré syndrome in adults.^5^ The major concern, however, arises when ZIKV infects pregnant women. The virus can cross the blood-placental barrier, preferentially targeting the fetal neuronal progenitor cells,^6^^,^^7^^,^^8^ thereby impacting brain development.^9^^,^^10^^,^^11^^,^^12^ ZIKV infection can cause microcephaly and other congenital abnormalities collectively referred to as Congenital Zika Syndrome (CZS).^13^^,^^14^^,^^15^ Since no vaccines or specific treatments are currently available, CZS remains a global health threat.

A recent study demonstrated that nicotinamide adenine dinucleotide (NAD^+^) metabolism is impaired in the brains of ZIKV-infected mouse embryos.^16^ The administration of nicotinamide riboside (NR), a precursor of NAD^+^, to pregnant mice carrying intracerebrally infected pups, followed by continued administration to their offspring after birth, significantly reduced neuronal cell death and partially prevented cortical thinning.^16^ These findings suggest that therapies aimed at preserving NAD^+^ homeostasis have the potential to mitigate ZIKV-induced neuronal damage. However, until now, the mechanisms underlying NAD^+^ metabolism disruption in this context remain unknown.

In addition to its role as a redox cofactor in energy metabolism, NAD^+^ serves as a substrate for enzymes such as PARPs, sirtuins, and NAD^+^ hydrolases, which regulate key cellular processes, including genome stability, epigenetic modifications, mitochondrial metabolism, and intracellular signaling.^17^ Because these reactions continuously consume NAD^+^, the activity of NAD^+^-degrading enzymes has profound consequences for cellular physiology, particularly in the nervous system, where NAD^+^ availability is closely linked to neuronal survival and immune responses.^17^

Building on this framework, emerging evidence indicates that NAD^+^-consuming enzymes play important roles in host-pathogen interactions. For instance, mono-ADP-ribosylating PARPs such as PARP12 are strongly induced by viral challenge and restrict viral replication by inhibiting protein translation, degrading viral RNA, and modifying viral proteins in infected cells.^18^ Other NADases, including SARM1, which promotes axonal degeneration through NAD^+^ cleavage, and CD157, implicated in leukocyte adhesion, migration, and inflammatory signaling, have also been linked to immune processes.^19^^,^^20^ The multifunctional ectoenzyme CD38, a major NADase in mammalian tissues,^21^ acts as a marker of immune cell activation and has been associated with bacterial, protozoan, and viral infections.^22^ In viral settings, increased CD38 expression is often observed on activated T lymphocytes and dendritic cells, and in some contexts has been linked to the modulation of antiviral responses and immune cell activation.^23^^,^^24^^,^^25^^,^^26^ However, whether CD38-mediated NAD^+^ depletion contributes directly to the immune response or viral pathogenesis remains poorly understood.

To counterbalance the continuous consumption of NAD^+^ by these enzymes, cells rely on biosynthetic pathways, among which the NAD^+^ salvage pathway predominates in most extra-hepatic tissues, including the brain.^27^ In this pathway, nicotinamide phosphoribosyltransferase (NAMPT) catalyzes the rate-limiting step by converting nicotinamide into NMN (nicotinamide mononucleotide), which is then used for NAD^+^ resynthesis. This central role places NAMPT as a key determinant of NAD^+^ availability under both physiological and pathological conditions. Indeed, NAMPT depletion is known to contribute to neurodegeneration in mice.^28^

Using an infection model developed by our group,^29^ here we conducted a thorough temporal analysis of NAD^+^ metabolism dynamics throughout the course of ZIKV infection in the brains of neonate mice. We found that NAD^+^ depletion is a late event in the infection and is likely an indirect consequence of the neuroinflammatory response that follows viral replication in the brain. Additionally, we observed that increased expression and activity of the NAD^+^-consuming enzyme CD38 – possibly driven by the infiltration of CD38^+^ immune cells into the brain – is the primary factor disrupting NAD^+^ homeostasis in this context. NAMPT expression was also upregulated during the course of infection, but this induction did not appear sufficient to counterbalance the NAD^+^ decline. By elucidating the mechanisms underlying NAD^+^ depletion, our findings provide a foundation for exploring therapeutic interventions targeting CD38 and NAD^+^ metabolism to mitigate the long-term effects of CZS.

## Results

### Nicotinamide adenine dinucleotide levels decrease at the latter stages of Zika virus replication in the brain

**Figure 1:**
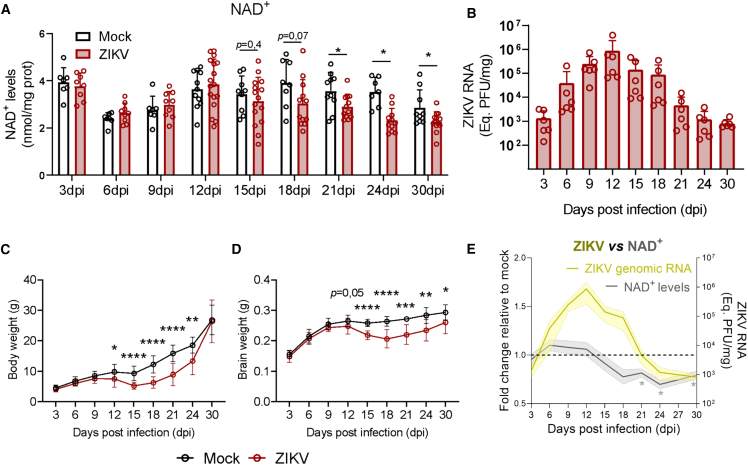
NAD^+^ levels decline in the brain at the late stages of ZIKV infection, following viral replication and disease onset (A) Total NAD^+^ levels in the brains of ZIKV-infected and mock-injected mice measured at different time points post-infection (dpi). Data show a progressive decline in NAD^+^ levels in ZIKV-infected animals starting at 18 dpi, with significant reductions observed at 21, 24, and 30 dpi (*n* ≥ 7 mice per group). (B) Quantification of ZIKV genomic RNA in brain tissue by absolute RT-qPCR, expressed as Log_10_-transformed PFU equivalents per mg of tissue (*n* = 6 mice per group). (C and D) Body weight and (D) brain weight of ZIKV-infected and mock-injected mice monitored throughout the course of infection (*n* ≥ 5 mice per group). (E) Overlay of ZIKV RNA (fold change relative to mock, yellow line, right y axis) and total NAD^+^ levels (fold change relative to mock, gray line, left y axis) throughout the infection timeline. The decline in NAD^+^ levels occurs after the peak of viral replication. Data in panels A–D are expressed as mean ± SD, and panel E as mean ± SEM (shaded area). Statistical significance refers to comparisons between Mock and ZIKV groups at each time point, and was performed using an unpaired Student’s *t* test or Mann-Whitney test, as appropriate. ∗*p* ≤ 0.05; ∗∗*p* ≤ 0.01; ∗∗∗*p* ≤ 0.001; ∗∗∗∗*p* ≤ 0.0001.

To investigate the temporal dynamics of NAD^+^ metabolism disturbances in the brains of ZIKV-infected mice, we subcutaneously infected postnatal day 3 (P3) mice with 2.7 x 10^5^ plaque-forming units (PFUs) of ZIKV, while control mice received mock injections. We euthanized mice every three days post-infection (dpi) up to 30 dpi, harvesting the brain to measure the NAD^+^ levels in this tissue. We observed that NAD^+^ levels in the brains of ZIKV-infected mice were significantly lower than those in mock controls at 18 dpi (0.78-fold, SD ± 0.26, *p* = 0.0727), 21 dpi (0.82-fold, SD ± 0,14, *p* = 0.0294), 24 dpi (0.66-fold, SD ± 0.14, *p* = 0.0003) and 30 dpi (0.79-fold, SD ± 0.15, *p* = 0.0277) (Figure 1A). Thus, NAD^+^ levels in the brain become dysregulated at the latter stages of ZIKV infection.

To address whether ZIKV replication kinetics align with the decline in NAD^+^ levels in the brain, we performed absolute quantitative PCR of ZIKV cDNA (equivalent to its genomic RNA) at different post-infection time points. ZIKV replication in the brain peaked at 12 dpi, followed by a progressive decline, reaching lower levels at 24 and 30 dpi (Figure 1B). Consistent with our previous findings,^29^ we observed signs of ZIKV disease from 12 dpi onwards, including reduced body and brain weights (Figures 1C and 1D). When we overlaid the fold change in NAD^+^ levels with the expression of equivalent PFU of ZIKV genomic RNA, it became evident that NAD^+^ levels significantly decreased at least one week after the peak of viral replication (Figure 1E). This suggests that the decrease in NAD^+^ levels is not a direct consequence of ZIKV replication but rather an indirect effect. Interestingly, this decline in NAD^+^ coincides with the time point when ZIKV-infected mice display the most pronounced reduction in body and brain weight, hinting at a potential link between NAD^+^ depletion and disease progression.

### The expression of non-conventional Parps correlates with viral replication but not with the decrease in NAD^+^ levels in the brain of ZIKV-infected mice

Next, we investigated potential mechanisms driving the decrease in NAD^+^ levels in the brains of ZIKV-infected mice, analyzing both NAD^+^ synthesis and consumption pathways. We first focused on NAD^+^-consuming enzymes. Since Parp12, a member of the Parp family of NAD^+^ hydrolases, has been described as playing a role in the innate immune response against ZIKV,^30^ we hypothesized that Parp12 could contribute to the decrease in NAD^+^ levels. To test this, we analyzed the time course of Parp12 transcriptional induction.

**Figure 2:**
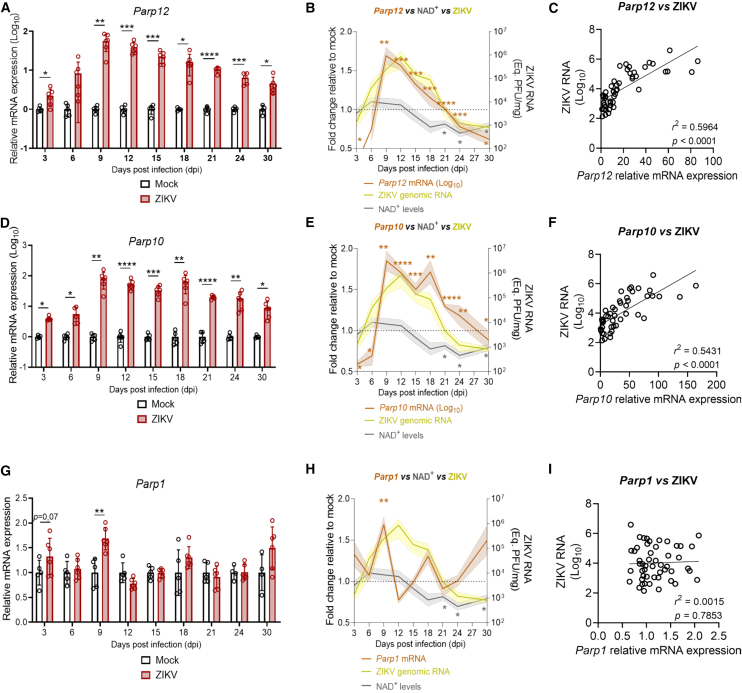
The induction of non-conventional PARPs correlates with ZIKV replication but not with the decline in NAD^+^ levels (A, D, G) Relative mRNA expression of *Parp12*, *Parp10*, and *Parp1* in the brains of ZIKV-infected and mock-injected mice across different time points post-infection (dpi). Both *Parp12* and *Parp10* were robustly induced during the early to mid-stages of infection (presented as log_10_-transformed relative values), whereas *Parp1* showed only a modest and transient induction. (B, E, H) Overlay of relative mRNA expression profiles of *Parp12* (B), *Parp10* (E), and *Parp1* (H), with total NAD^+^ levels (gray line) and ZIKV genomic RNA (yellow line), showing that *Parp10* and *Parp12* induction coincides with viral replication but precedes the decline in NAD^+^ levels. (C, F, I) Linear regression analyses show a strong correlation between ZIKV genomic RNA and *Parp12* (C) and *Parp10* (F) mRNA expression, but no significant correlation with *Parp1* expression (I). Data in A, D, and G are presented as mean ± SD; in B, E, and H as mean ± SEM (shaded area). Statistical significance was determined using an unpaired Student’s *t* test or Mann-Whitney test, as appropriate. *n* ≥ 4 mice per group (A, D, G); *n* = 49 (C), *n* = 48 (F), *n* = 51 (I). ∗*p* ≤ 0.05; ∗∗*p* ≤ 0.01; ∗∗∗*p* ≤ 0.001; ∗∗∗∗*p* ≤ 0.0001.

We observed a robust increase in *Parp12* mRNA expression from 3 dpi to 12 dpi compared to mock controls (2.2-fold, SD ± 1.07, *p* = 0.0307 at 3 dpi; 8.29-fold, SD ± 7.83, *p* = 0.0695 at 6 dpi, 55.7-fold, SD ± 26.1, *p* = 0.0011 at 9 dpi; 38.5-fold, SD ± 13.5, *p* = 0.0001 at 12 dpi; Figure 2A). After this peak, *Parp12* mRNA levels progressively declined, but remained 4.5 times higher than mock controls at 30 dpi (SD ± 2.15, *p* = 0.0128; Figure 2A). When we overlaid the *Parp12* expression curve with the fold change in NAD^+^ levels and the genomic ZIKV replication, we found that the peak of *Parp12* induction closely followed ZIKV replication in the brain and preceded the decrease in NAD^+^ levels by at least one week (Figure 2B). Moreover, we detected a strong correlation between ZIKV genome levels and *Parp12* expression in this tissue (*r*^*2*^ = 0.5964, *p* < 0.0001; Figure 2C). These results suggest that the late-stage decrease in NAD^+^ levels in ZIKV-infected mice is not driven by Parp12 activity.

We also analyzed the expression dynamics of *Parp10*, another non-conventional Parp involved in antiviral responses,^31^^,^^32^ and *Parp1*, whose activity has been linked to NAD^+^ depletion in other contexts.^33^ The induction pattern of *Parp10* mRNA in ZIKV-infected brains closely resembled that of *Parp12*, both in timing and intensity (Figures 2D and 2E). However, *Parp1* exhibited only a 1.7-fold induction (SD ± 0.23, *p* = 0.0015) at 9 dpi, with no significant changes at other time points (Figures 2G and 2H). While *Parp10* expression was significantly correlated with ZIKV RNA levels (Figures 2E and 2F), *Parp1* mRNA induction did not (Figures 2H and 2I). Finally, the peak expression of *Parp10*, *Parp12*, and *Parp1* mRNA did not coincide with the decrease in NAD^+^ levels in the brains. Thus, similarly to *Parp12*, neither *Parp10* nor *Parp1* appears to be the main driver of NAD^+^ metabolism imbalance in this tissue. Altogether, the expression of the non-conventional Parps follows ZIKV replication in the brains of infected mice but does not explain the decrease in NAD^+^ levels, which occurs later in the infection.

### The expression and activity of CD38 closely correlate with the decrease in nicotinamide adenine dinucleotide levels in the brain of Zika virus-infected mice

**Figure 3:**
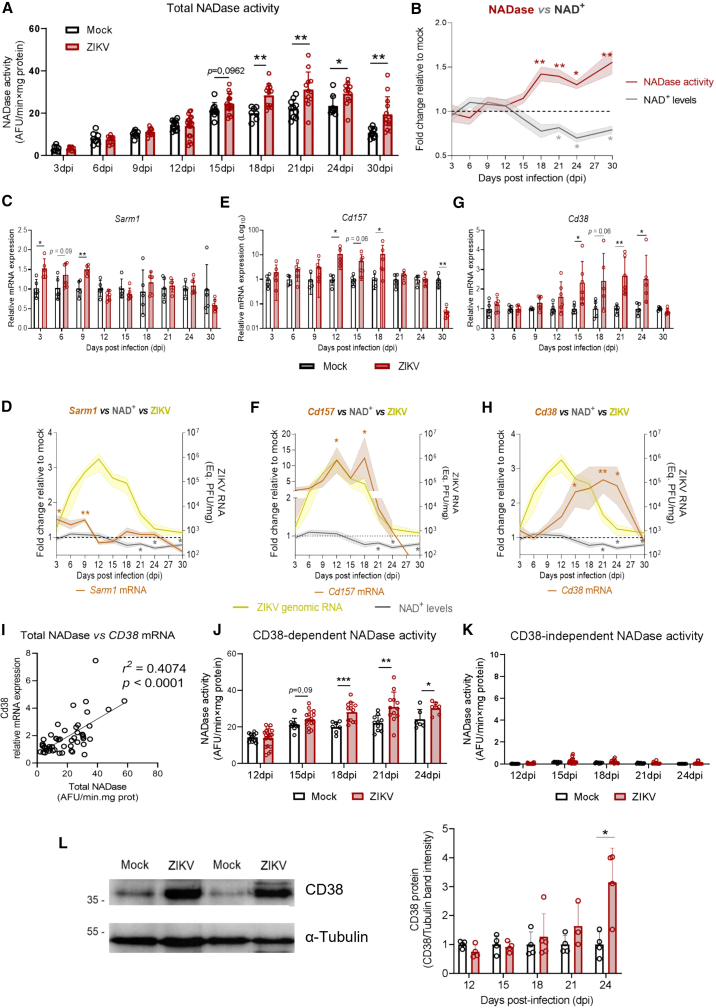
CD38 expression and activity increase at late stages of ZIKV infection and correlate with NAD^+^ decline in the brain (A) Total NADase activity measured in the brains of ZIKV-infected and mock-injected mice over the course of infection, showing significant increases from 18 dpi onward (*n* ≥ 7 mice per group). (B) Overlay of NADase activity (red line) and NAD^+^ levels (gray line), both relative to mock controls (dashed line), showing an inverse temporal association. (C–H) Relative mRNA expression of *Sarm1*, *Cd157*, and *Cd38*, respectively, in the brains of ZIKV-infected and control mice (*n* ≥ 4 mice per group). (D, F, H) Overlays of mRNA expression profiles of *Sarm1* (D), *Cd157* (F), and *Cd38* (H) with NAD^+^ levels (gray line) and ZIKV genomic RNA (yellow line), indicating that *Cd38* induction temporally coincides with NAD^+^ decline, while *Sarm1* and *Cd157* do not. (I) Linear regression shows a positive correlation between total NADase activity and *Cd38* mRNA expression (*n* = 50). (J) CD38-dependent NADase activity, calculated as the fraction inhibited by the specific CD38 inhibitor 78c (*n* ≥ 6 mice per group). (K) CD38-independent NADase activity, which remains low and unchanged during infection (*n* ≥ 6 mice per group). (L) On the right, representative Western blot of CD38 protein expression in brain extracts at 24 dpi, with α-tubulin as loading control (representative bands from the same experiment shown in the full blot in Figure S1). On the left, quantification of the Western blot bands’ intensities (CD38/α-tubulin) relative to mock (*n* ≥ 4 mice per group). Data in panels A, C, E, G, J, K, and L are presented as mean ± SD; panels B, D, F, and H as mean ± SEM (shaded area). Statistical significance was determined by unpaired Student’s *t* test or Mann–Whitney test, as appropriate. ∗*p* ≤ 0.05; ∗∗*p* ≤ 0.01; ∗∗∗*p* ≤ 0.001.

Since Parp induction did not coincide with the decrease in NAD^+^ levels in the brains of ZIKV-infected mice, we investigated whether NAD^+^-glycosyl hydrolases could play a role in this process, as this class of enzymes has been implicated in NAD^+^ depletion in other contexts.^34^^,^^35^ To explore this possibility, we measured the total activity of NAD^+^-glycosyl hydrolases (hereafter referred to as NADases) at different post-infection time points. We observed a significant increase in NADase activity in the brains of ZIKV-infected mice compared to mock controls at 18 dpi (1.42-fold, SD ± 0.27, *p* = 0.0017), 21 dpi (1.4-fold, SD ± 0.37, *p* = 0.0063), 24 dpi (1.25-fold, SD ± 0.22, *p* = 0.0314), and 30 dpi (1.81-fold, SD ± 0.76, *p* = 0.0054) (Figure 3A). This increase in NADase activity closely coincided with the decline in NAD^+^ levels in the brains of ZIKV-infected mice (Figure 3B), suggesting that it may be driving NAD^+^ depletion in the brain during infection.

To identify which NADases contribute to the loss of NAD^+^ homeostasis during ZIKV infection, we analyzed the mRNA expression of *Sarm1*, *Cd38*, and *Cd157* in the brains of ZIKV-infected neonate mice over the course of infection. Both *Sarm1* and *Cd157* were transcriptionally induced in the brains of ZIKV-infected mice, but did not exhibit expression patterns that matched the decline in NAD^+^ levels. *Sarm1* was upregulated at early time points, while *Cd157* expression increased between 12 and 18 dpi, largely paralleling ZIKV genomic RNA levels. However, neither showed a temporal correlation with NAD^+^ depletion (Figures 3C–3F). Among the analyzed NADases, only *Cd38* mRNA expression significantly increased at later time points, from 15 to 24 dpi (2.33-fold, SD ± 1.08, *p* = 0.0254 at 15 dpi; 2.43-fold, SD ± 1.4, *p* = 0.0606 at 18 dpi; 2.7-fold, SD ± 0.94, *p* = 0.0037 at 21 dpi; and 2.5-fold, SD ± 1.225, *p* = 0.0279 at 24 dpi; Figure 3G), closely aligning with the decrease in NAD^+^ levels in this tissue (Figure 3H). At this stage, transcript-level data pointed to CD38 as a potential mediator of NAD^+^ depletion in the brains of ZIKV-infected mice. To explore whether *Cd38* mRNA induction during infection translates into increases in CD38 activity and protein expression in the brain, we assessed NADase activity and CD38 protein levels in the brains of ZIKV-infected mice. We found a moderate correlation between *Cd38* mRNA induction and total NADase activity (*r*^*2*^ = 0.4074, *p* < 0.001; Figure 3I), suggesting that CD38 is the primary NADase in this context.

To further confirm the contribution of CD38, we added 78c, a specific reversible, uncompetitive inhibitor of CD38,^36^ to the reaction media of the NAD^+^ glycosyl-hydrolase activity assay to determine the fraction of CD38-dependent activity in brain protein lysates. This approach revealed that: (a) by comparing total NAD^+^ glycosyl-hydrolase activity in the absence of inhibitor with the residual activity in the presence of the specific CD38 inhibitor 78c, we found that ∼99% of the total activity is CD38-dependent, even in mock controls; (b) CD38 activity significantly increased at 18 dpi (1.41-fold, SD ± 0.27, *p* = 0.0017), 21 dpi (1.39-fold, SD ± 0.37, *p* = 0.0063), and 24 dpi (1.25-fold, SD ± 0.13, *p* = 0.0292) (Figure 3J); (c) CD38-independent NADase activity represents only a minor fraction of total NAD^+^ glycosyl-hydrolase activity and is not significantly induced during ZIKV infection (Figure 3K). In addition to increased CD38 activity, we observed a significant induction in CD38 protein expression at 24 dpi (3.16-fold, SD ± 1.17, *p* = 0.0135; Figure 3L, see also Figure S1). Therefore, CD38 is the main NADase expressed at later stages of ZIKV infection in the brain and likely drives the disturbance in NAD^+^ levels in this context.

### CD38 inhibition prevents the Zika virus-induced decrease in nicotinamide adenine dinucleotide levels in the brain

**Figure 4:**
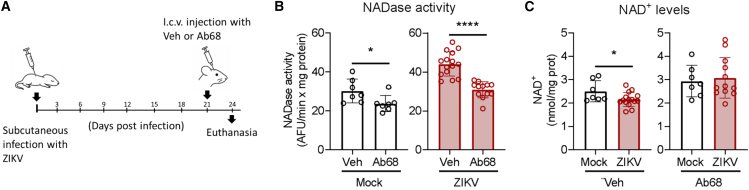
CD38 inhibition prevents NAD^+^ depletion in the brains of ZIKV-infected mice (A) Schematic representation of the experimental design. Neonatal mice were subcutaneously infected with ZIKV at postnatal day 3 (P3). At 21 days post-infection (dpi), animals received a unilateral intracerebroventricular (i.c.v.) injection of the CD38-blocking antibody Ab68 (5.76 μg) or vehicle (Veh), and brains were collected at 24 dpi for analysis. (B) NAD^+^ hydrolase activity in brain tissue. (C) Quantification of total NAD^+^ levels in brain tissue. Data are presented as mean ± SD. Statistical analyses were performed using an unpaired Student’s *t* test (*n* ≥ 7 mice per group). ∗p ≤ 0.05; ∗∗∗∗p ≤ 0.0001.

In a pilot experiment, ICV injection of the small-molecule inhibitor 78c failed to alter brain NAD^+^ levels, which may reflect limited penetration or stability in the CNS. In contrast, Ab68, an antibody that blocks CD38 activity, provides potent and sustained CD38 inhibition *in vivo*, with a single injection suppressing activity for several days, including in collected tissues.^37^ Therefore, to confirm whether CD38 modulates the NAD^+^ levels in the brain of ZIKV-infected neonate mice, we infected P3 mice and performed an intracerebroventricular injection of Ab68 at 21 dpi. Three days later, we euthanized the mice and collected their brains for analysis (Figure 4A). We confirmed that i.c.v. injection of Ab68 significantly reduced NAD^+^ hydrolase activity in the brains of both mock- and ZIKV-infected mice (Figure 4B). In vehicle-treated mice, ZIKV infection reduced NAD^+^ levels by 14% (SD ± 0.12, *p* = 0.0450) compared to mock controls (Figure 4C). However, in the presence of Ab68, NAD^+^ levels remained unchanged (2.94 nmol/mL ± 0,67 in mock vs. 3.08 nmol/mL ± 0.87 in ZIKV-infected mice, *p* = 0.7106). These findings suggest that CD38 is the main NAD^+^-consuming enzyme responsible for the NAD^+^ depletion in the brains of ZIKV-infected mice.

### Nicotinamide phosphoribosyltransferase is induced early during infection but fails to prevent late nicotinamide adenine dinucleotide depletion

**Figure 5:**
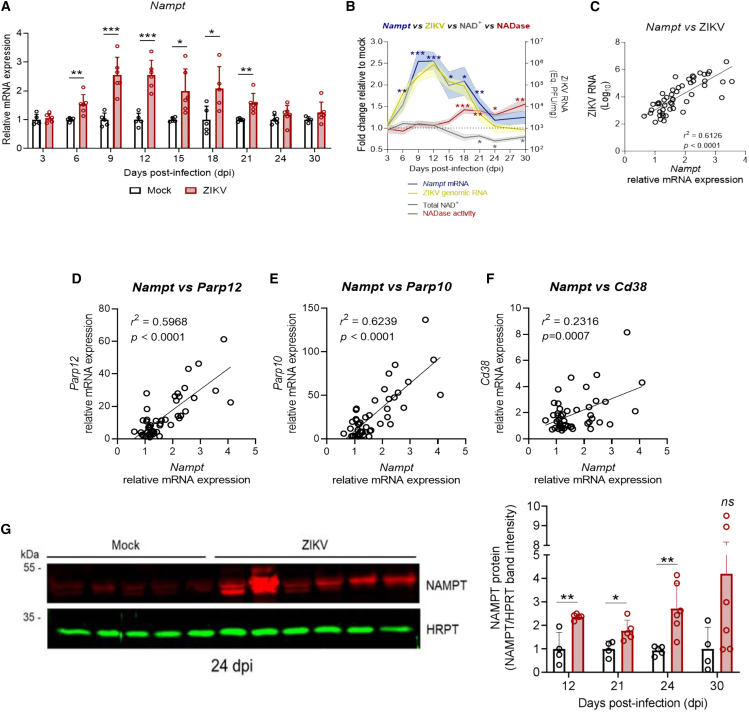
NAMPT is induced in the brain of ZIKV-infected mice (A) Relative mRNA expression of *Nampt* in the brains of ZIKV-infected and mock-injected mice across time points post-infection (*n* ≥ 4 mice per group). (B) Overlay of *Nampt* mRNA expression (blue line), total NADase activity (red line), NAD^+^ levels (gray line), and ZIKV genomic RNA (yellow line), all relative to mock controls (dashed line). (C–F) Correlation analyses between *Nampt* mRNA expression and (C) ZIKV genomic RNA, (D) *Parp12*, (E) *Parp10*, and (F) *Cd38* mRNA expression. *Nampt* shows a strong correlation with viral load and early-induced *Parps*, but a weak correlation with *Cd38*. (G) Representative Western blot of NAMPT protein expression in the brains of ZIKV-infected and mock-injected mice at 24 days post-infection (dpi; representative bands from the same experiment shown in the full blot in Figure S2).. HPRT was used as a loading control. The right panel shows quantification of NAMPT protein levels (NAMPT/HPRT ratio), normalized to mock controls (*n* ≥ 4 mice per group). Data in panels A and G are presented as mean ± SD; panel B as mean ± SEM (shaded area). Correlations were assessed by linear regression analysis. Statistical analyses were performed using an unpaired Student’s *t* test or Mann–Whitney test, as appropriate. ∗*p* ≤ 0.05; ∗∗*p* ≤ 0.01; ∗∗∗*p* ≤ 0.001.

To investigate whether disruptions in NAD^+^ synthesis also contribute to the NAD^+^ imbalance observed in the brains of ZIKV-infected mice, we analyzed changes in the expression of Nampt, the enzyme that catalyzes the rate-limiting step of the NAD^+^ salvage pathway—the predominant NAD^+^ biosynthetic route in extrahepatic tissues, including the brain.^27^ *Nampt* transcriptional expression increased in the brains of ZIKV-infected mice compared to mock controls as early as 6 dpi (1.53-fold, SD ± 0.34, *p* = 0.0069), rising to 2.49-fold (SD ± 0.62, *p* = 0.0005) at 9 dpi, and remaining at similar levels until 15 dpi (2.39-fold, SD ± 0.51, *p* = 0.0001 at 12 dpi; 2.14-fold, SD ± 0.77, *p* = 0.02 at 15 dpi; Figure 5A). After this peak, *Nampt* expression began to decline, reaching 1.79-fold (SD ± 0.75, *p* = 0.0253) at 18 dpi and 1.57-fold at 21 dpi (SD ± 0.34, *p* = 0.0048), before returning to baseline levels from 24 dpi onward (Figure 5A). Thus, the pattern of *Nampt* induction closely follows ZIKV replication in the brain but does not align with the later induction of NADases or the decrease in NAD^+^ levels during infection (Figure 5B). Indeed, *Nampt* expression strongly correlated with ZIKV genome levels (*r*^2^ = 0.6126, *p* < 0.0001; Figure 5C). Additionally, *Nampt* expression in the infected mice showed a strong correlation with *Parp12* (*r*^2^ = 0.5968, *p* < 0.0001; Figure 5D) and *Parp10* (*r*^2^ = 0.6239, *p* < 0.0001; Figure 5E) but displayed only a weak correlation with *Cd38* induction (*r*^2^ = 0.2316, *p* = 0.0007; Figure 5F).

Finally, we confirmed that NAMPT protein expression is upregulated in the brains of ZIKV-infected mice, reaching 2.4-fold higher levels at 12 dpi (SD ± 0.12, *p* = 0.0031), coinciding with the peak of ZIKV genome replication. Notably, NAMPT protein levels remained elevated at later stages of infection, with a 1.77-fold increase at 21 dpi (SD ± 0.45, *p* = 0.0237) and a 2.93-fold increase at 24 dpi (SD ± 2.57, *p* = 0.0052) compared to mock controls (Figure 5G, see also Figure S2). At 30 dpi, NAMPT expression also remained elevated (4.2-fold on average), although the difference was not statistically significant due to high inter-sample variability (SD ± 3.96, *p* = 0.1583). These findings suggest that NAMPT protein levels do not strictly mirror its transcriptional profile, possibly due to post-translational mechanisms that stabilize the protein during the later stages of infection, when cellular demand for NAD^+^ salvage may be highest. Additionally, NAMPT protein levels at later stages of infection remain comparable to those observed at 12 dpi, with no further upregulation.

Altogether, our findings suggest that NAMPT expression is upregulated during the peak of ZIKV replication in the brain, coinciding with the induction of the NAD^+^-consuming enzymes *Parp10* and *Parp12*, and remains elevated during the later stages of infection, when CD38 expression and activity increase. However, this sustained activation of the NAD^+^ salvage pathway appears insufficient to counterbalance the heightened NAD^+^ consumption at these later time points, as evidenced by the decline in NAD^+^ levels. Nevertheless, NAMPT upregulation may play a role in maintaining NAD^+^ homeostasis during the early stages of infection, potentially buffering the increased NAD^+^ demand driven by Parp activation.

### Neuroinflammation precedes CD38 induction in Zika virus-infected mice

**Figure 6:**
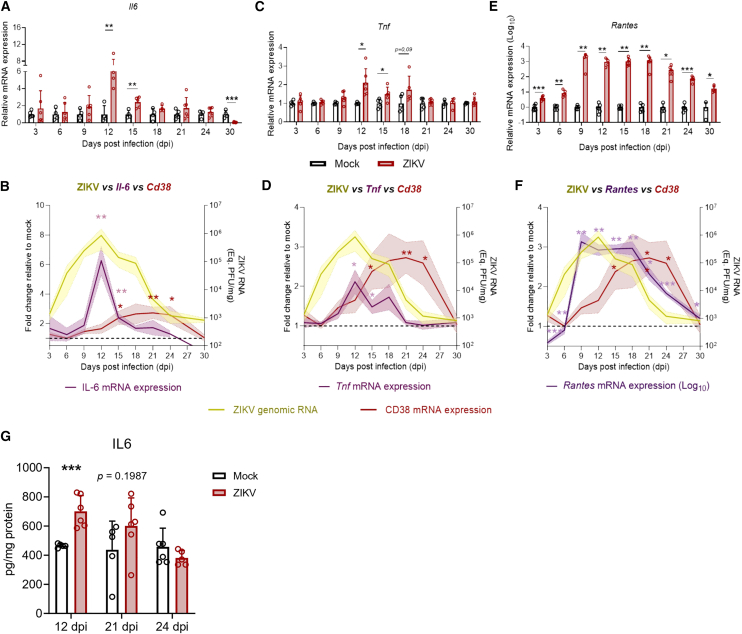
Proinflammatory cytokine expression precedes CD38 induction in the brains of ZIKV-infected mice (A, C, E) Relative mRNA expression of *Il6* and *Tnf* (linear scale), and *Ccl5/Rantes* (Log_10_-transformed) in the brains of ZIKV-infected and mock-injected mice across time points post-infection (*n* ≥ 3 mice per group). All three inflammatory mediators were significantly upregulated during the early and mid-stages of infection. (B, D, F) Overlay of *Il6* (B), *Tnf* (D), and *Rantes* – log_10_ scale (F) mRNA expression profiles (purple line) with *Cd38* mRNA (red line), NAD^+^ levels (gray line), and ZIKV genomic RNA (yellow line), all relative to mock controls (dashed line). (G) Brain IL-6 protein levels determined by ELISA (*n* ≥ 5 mice per group). The temporal pattern shows that cytokine and chemokine induction precede *Cd38* expression, suggesting that neuroinflammation may contribute to the upregulation of CD38. Data in panels A, C, and E are presented as mean ± SD; panels B, D, and F as mean ± SEM (shaded area). Statistical analyses were performed using an unpaired Student’s *t* test or Mann–Whitney test, as appropriate. ∗*p* ≤ 0.05; ∗∗*p* ≤ 0.01; ∗∗∗*p* ≤ 0.001.

Since CD38 is known to be induced by inflammatory mediators, such as cytokines and chemokines,^38^^,^^39^^,^^40^ we investigated whether CD38 induction could result from ZIKV-induced neuroinflammation. To address this, we analyzed the transcriptional expression of the cytokines *Il-6*, *Tnf*, and the chemokine *Ccl5*/*Rantes* throughout the course of infection in the brain. We observed an induction of *Il-6*, with a peak at 12 dpi (7.4-fold, SD ± 2.6, *p* = 0.0025), followed by a significant decline at 15 dpi (2.83-fold compared to mock, SD ± 0.73, *p* = 0.0032) and a return to baseline levels by 18 dpi (Figure 6A). When we overlaid the *Il-6* and *Cd38* fold-change expression curves with ZIKV genomic RNA levels, it became evident that the peak of *Il-6* induction coincided with the peak of viral replication in the brain and preceded *Cd38* induction (Figure 6B). We also observed a moderate induction of *Tnf* in the brains of ZIKV-infected mice, with a 2.13-fold increase at 12 dpi (SD ± 0.75, *p* = 0.018), a 1.49-fold increase at 15 dpi (SD ± 0.39, *p* = 0.0422), and a return to the baseline levels by 21 dpi (Figure 6C). Similarly to *Il-6*, the peak of *Tnf* induction coincided with ZIKV replication and preceded *Cd38* induction in the brain (Figure 6D).

*Ccl5/Rantes* transcriptional induction occurred early after infection, with a 4.11-fold increase at 3 dpi (SD ± 1.29, *p* = 0.0007), a 9.08-fold increase at 6 dpi (SD ± 4.64, *p* = 0.0046), followed by a massive peak at 9 dpi (2,032.2-fold, SD ± 1,129.26, *p* = 0.0038; Figure 6E). *Ccl5/Rantes* levels remained elevated, sustaining around 1,000-fold induction from 12 to 18 dpi (1,015.78-fold, SD ± 605.84, *p* = 0.0028 at 12 dpi; 1,087.45-fold, SD ± 688.06, *p* = 0.0043 at 15 dpi; 1,273.14-fold, SD ± 762.13, *p* = 0.0049 at 18 dpi) before declining progressively to 289.1-fold (SD ± 219.59, *p* = 0.0331) at 21 dpi, 75.7-fold (SD ± 27.06, *p* = 0.0002) at 24 dpi and ultimately 16.8-fold (SD ± 8.00, *p* = 0.0132) at 30 dpi (Figure 6E). Thus, unlike *Il-6* and *Tnf*, the peak of *Ccl5/Rantes* induction occurs before the peak of viral replication and remains elevated even after ZIKV genomic RNA levels decline in the brain (Figure 6F). However, similarly to *Il-6* and *Tnf*, *Ccl5/Rantes* induction precedes *Cd38* induction in the brains of ZIKV-infected mice (Figure 6F). Consistent with the transcriptional data, IL-6 protein levels increased at 12 dpi and returned to baseline by 21–24 dpi (Figure 6G). Therefore, CD38 induction may be driven by increased inflammatory mediators in the brain.

### Infiltrating CD38-positive immune cells accumulate in the brain at the later stages of Zika virus infection

Among the inflammatory mediators induced by ZIKV infection in the brain, the chemokine *Ccl5*/*Rantes* showed the highest expression, suggesting a key role in recruiting immune cells to this tissue. Therefore, we next investigated whether the increase in CD38 expression and activity in the brains of ZIKV-infected mice could result from the infiltration of CD38^+^ immune cells. To address this, we isolated immune cells from the brains of ZIKV-infected and mock-injected mice at the time point corresponding to the most significant decline in NAD^+^ levels (24 dpi), using a density gradient centrifugation protocol.

**Figure 7:**
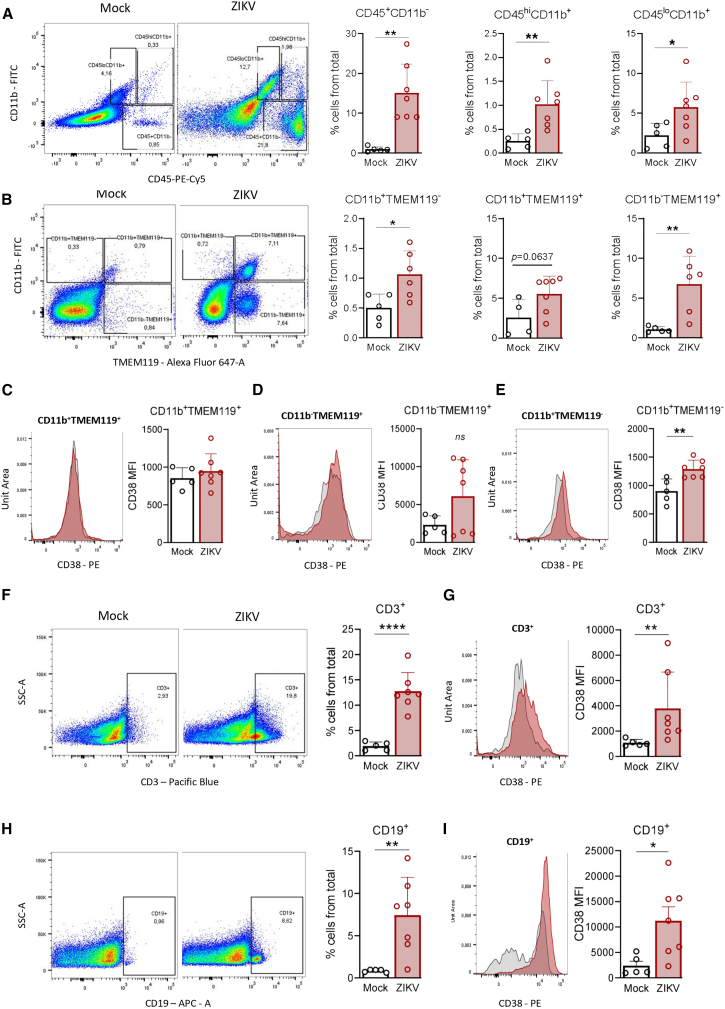
Infiltrating immune cells contribute to increased CD38 expression in the brains of ZIKV-infected mice (A) On the left, representative dot plots show the gating strategy used to identify CD45^lo^CD11b^+^ (putative resting microglia), CD45^hi^CD11b^+^ (infiltrating myeloid cells), and CD45^+^CD11b^−^ (lymphoid cells) populations. On the right, graphs showing the frequency of each population in ZIKV-infected and mock-injected mice (*n* ≥ 5 mice per group). (B) Representative dot plots and quantification of CD11b^+^TMEM119^+^, CD11b^+^TMEM119^+^, and CD11b^+^TMEM119^-^ populations in the brains of infected and control mice (*n* ≥ 6 mice per group), with the respective graphs showing the frequency of each population. (C–E) Representative histograms and quantification of CD38 expression (median fluorescence intensity, MFI) in CD11b^+^TMEM119^+^ (C) CD11b^−^TMEM119^+^ (D), and CD11b^+^TMEM119^-^ (E) populations. (F and G) Gating and quantification of CD3^+^ T cells (F) and corresponding CD38 expression (G). (H and I) Gating and quantification of CD19^+^ B cells (H) and corresponding CD38 expression (I). All graphs represent mean ± SD. Gating was based on negative controls; histogram quantification was performed using MFI, and curves were normalized to unit area. Statistical analyses were performed using an unpaired Student’s *t* test or Mann–Whitney test, as appropriate. ∗*p* ≤ 0.05; ∗∗*p* ≤ 0.01; ∗∗∗∗*p* ≤ 0.0001.

We first used antibodies against the surface markers CD45 and CD11b to label and identify three distinct immune cell populations. The CD45^+^CD11b^−^ population, which includes lymphoid cells such as T and B lymphocytes and natural killer (NK) cells, showed a marked increase in ZIKV-infected brains compared to mock controls (16.18-fold, SD ± 7.67, *p* = 0.0014; Figure 7A). The CD45^hi^CD11b^+^ population, corresponding to infiltrating macrophages, was also significantly increased (4.02-fold, SD ± 1.94, *p* = 0.0076). We also observed significant changes in the CD45^lo^CD11b^+^ population (2.57-fold, SD ± 1.42, *p* = 0.0461), which is consistent with tissue-resident microglia (Figure 7A). These findings indicate a robust inflammatory infiltrate in the brains of ZIKV-infected mice, predominantly composed of lymphoid cells, along with microgliosis.

The CD45^lo^CD11b^+^ phenotype alone is insufficient to reliably distinguish microglia from infiltrating myeloid cells, as activated microglia can upregulate CD45 expression under inflammatory conditions.^41^ To overcome this limitation, we used an antibody against TMEM119, a microglia-specific marker, allowing us to differentiate TMEM119^+^ microglia from CD11b^+^TMEM119^-^ infiltrating macrophages. CD11b^+^TMEM119^-^, CD11b^+^TMEM119^+^, and CD11b^−^TMEM119^+^ cell populations were all increased in the brains of ZIKV-infected mice compared to mock controls (2.12-fold, SD ± 0.78, *p* = 0.0203; 2.17-fold, SD ± 0.87, *p* = 0.0637; and 6.45-fold, SD ± 3.37, *p* = 0.0061, respectively; Figure 7B), indicating that both macrophage infiltration and microgliosis—a phenomenon previously described in ZIKV neuroinfection^29^^,^^42^^,^^43^ —occur in the brains of ZIKV-infected mice.

We then assessed CD38 expression in both CD11b^+^TMEM119^+^ and CD11b^−^TMEM119^+^ microglial populations; however, neither exhibited a substantial increase in CD38 expression (Figures 7C and 7D). Therefore, microglia are unlikely to be a major contributor to the overall CD38 upregulation observed in the brain. In contrast, CD11b^+^TMEM119^-^ cells, corresponding to infiltrating macrophages, showed a significant increase in CD38 expression (1.43-fold, SD ± 0.17, *p* = 0.0042; Figure 7D), supporting the idea that macrophages may contribute to the NAD^+^ depletion during the late stages of infection.

Likewise, to further characterize the lymphoid compartment, we used antibodies against CD3 and CD19 to identify total T and B cell populations, respectively. In ZIKV-infected brains, CD3^+^ T cells were significantly increased (6.66-fold, SD ± 1.93, *p* < 0.0001; Figure 7F), accompanied by a notable rise in CD38 expression within this population (3.53-fold, SD ± 2.67, *p* = 0.0638; Figure 7G). CD19^+^ B cells were also markedly elevated (8.76-fold, SD ± 5.28, *p* = 0.0089; Figure 7H), with a concurrent and statistically significant increase in CD38 expression (4.64-fold, SD ± 2.96, *p* = 0.0243; Figure 7I). These findings indicate that the lymphocytic infiltrate in ZIKV-infected brains contributes to CD38 upregulation during infection.

## Discussion

While a previous study reported disruptions in NAD^+^ homeostasis during ZIKV infection,^16^ the molecular mechanisms underlying this imbalance remain unclear. Here, we demonstrate that the decrease in NAD^+^ levels in the brain of ZIKV-infected mice is a late-stage event, occurring nearly one week after the peak of viral replication, and coincides with increased CD38 expression, enhanced CD38 activity, and the infiltration of CD38^+^ immune cells into the brain. We provide the first evidence that CD38 is a key driver of NAD^+^ depletion in this context, establishing a direct link between neuroinflammation and metabolic dysfunction in ZIKV-infected mice.

Loss of NAD^+^ homeostasis has been associated with multiple conditions, including neurodegeneration, metabolic dysfunctions, and aging-related diseases.^44^ Nonetheless, only a few studies have investigated the role of NAD^+^ imbalances in the pathophysiology of viral diseases. Here, we conducted a comprehensive analysis of NAD^+^ level dynamics and associated pathways throughout the course of ZIKV infection in the brain to gain insights into the mechanisms underlying NAD^+^ metabolic disruption in this condition.

One of the earliest metabolic events observed in ZIKV-infected brains is the robust induction of *Parp10* and *Parp12*, which codify for the non-conventional PARPs, enzymes that consume NAD^+^ to catalyze mono-ADP-ribosylation of proteins.^45^ PARPs are known to play antiviral roles, with PARP12 promoting ADP-ribosylation and proteasomal degradation of ZIKV non-structural proteins NS1 and NS3,^30^ a process further enhanced by PARP11.^46^ Thus, PARP12 likely contributes to limiting ZIKV dissemination within the infected brain. Similarly, we found that *Parp10* is also highly induced, paralleling *Parp12* expression. Since PARP10 restricts the replication of other viruses, including Chikungunya virus and avian influenza virus,^31^^,^^47^ it may exert a similar antiviral effect against ZIKV. However, further studies are needed to confirm this hypothesis. Importantly, neither *Parp10* nor *Parp12* appears to drive the late-stage NAD^+^ depletion observed in ZIKV-infected brains, as their expression returns to baseline levels over time. Instead, the early and coordinated induction of both *Parps* and *Nampt* suggests that the NAD^+^ salvage pathway may be upregulated to support NAD^+^ synthesis from precursors and buffer Parp-mediated NAD^+^ consumption during the initial phase of infection.

On the other hand, NAMPT protein expression remains elevated during the later stages of infection and does not increase further alongside the rise in CD38 expression and activity, suggesting that NAMPT induction alone is insufficient to prevent the NAD^+^ decline in ZIKV-infected brains. Alternatively, NAMPT may not be upregulated within the same cell populations that express CD38. CD38 is an ectoenzyme with glycosyl-hydrolase activity that uses NAD^+^ as a substrate to generate second messengers such as ADPR, cADPR, and NAADP, which regulate intracellular calcium fluxes for signaling purposes.^22^ In addition, it modulates nicotinamide levels under physiological conditions.^48^ Notably, CD38 is considered the primary NAD^+^-consuming enzyme *in vivo*, and its activity has been implicated in NAD^+^ depletion across multiple pathological conditions.^21^^,^^22^ However, in the mouse brain, the extent to which CD38 dictates NAD^+^ levels remains unclear.^49^ In our study, CD38 upregulation emerges as a late-stage event during ZIKV infection in the brain, coinciding with the decline in NAD^+^ levels, but not with the peak of viral replication. Inhibition of CD38 with Ab68 effectively prevented infection-induced NAD^+^ depletion, supporting the notion that CD38 is the main driver of this process in the brains of ZIKV-infected mice. However, we cannot rule out the possibility that the cumulative activity of other NAD^+^-consuming enzymes, such as PARPs, may also contribute to this phenomenon.

Our data reveal a temporal dissociation between CD38 mRNA upregulation, protein induction, and enzymatic activity during ZIKV infection. While CD38 protein levels showed only a modest increase and reached statistical significance at 24 dpi, enzymatic activity began to rise as early as 15 dpi and was significantly elevated from 18 dpi onwards. This temporal pattern suggests that post-translational mechanisms may enhance CD38 activity before detectable changes in total protein abundance. Notably, the period of highest activity also coincides with the most pronounced NAD^+^ depletion, underscoring its functional relevance. Flow cytometry analyses—although not encompassing all potential CD38-expressing cell types (e.g., endothelial cells)—identified a likely contribution from resident microglia as well as infiltrating macrophages and lymphocytes at 24 dpi, when protein levels also increase. Together, these findings suggest two overlapping events: an early activity increase driven mainly by resident brain cells, followed by a later protein upregulation at 24 dpi, potentially reflecting immune cell infiltration.

Inflammatory responses have been linked to metabolic disruptions, including NAD^+^ depletion. CD38 is known to be upregulated in inflammatory conditions,^22^^,^^38^ raising the hypothesis that neuroinflammation during ZIKV infection could contribute to NAD^+^ imbalance. Here, we show that CD38 induction occurred days to weeks after the peak expression of *Il-6*, *Tnf*, and *Ccl5*/*Rantes* in the brains of ZIKV-infected mice. Notably, all of these inflammatory mediators have been reported to strongly induce CD38 expression in macrophages, microglia, and endothelial cells.^39^^,^^50^ However, CD38 activity was not induced immediately following cytokine upregulation, but rather days to weeks later, suggesting that its expression is not primarily driven by resident brain cells. Instead, the sustained high levels of the chemotactic factor *Ccl5*/*Rantes* preceding the increase in CD38 led us to hypothesize that CD38 protein upregulation at 24 dpi results from the accumulation of infiltrating CD38^+^ immune cells in the infected brain.

Perivascular inflammation accompanied by lymphocytic infiltration in the central nervous system has been previously described in cases of congenital ZIKV infection in humans, macaques, and mouse models, supporting the idea of a local adaptive immune response linked to neuropathogenesis.^51^^,^^52^^,^^53^ In line with these findings, we observed CD3^+^ (T lymphocytes) and CD19^+^ (B lymphocytes) infiltrating the brains of ZIKV-infected mice. Notably, both populations showed elevated CD38 expression at 24 dpi—a time point marked by a significant decline in brain NAD^+^ levels and corresponding to the humoral phase of the immune response. Given the high CD38 expression in antibody-secreting cells such as plasmablasts and plasma cells,^54^^,^^55^^,^^56^ it is plausible that a portion of these CD19^+^ cells represents differentiating B cells actively contributing to the increased CD38 activity. However, as CD19 is often downregulated in terminally differentiated plasmocytes^57^ and CD38 can also be expressed by activated non-secretory B cells, further phenotyping with markers such as CD138 will be necessary to define this population more precisely.

Our data also demonstrate a significant accumulation of CD3^+^ T lymphocytes in the brains of ZIKV-infected mice, with the increased expression of CD38 within this population. This finding suggests a robust T cell response, possibly driven by local antigen presentation or by the disruption of the blood-brain barrier. Previous studies have shown that CD8^+^ effector T cells can play a dual role in ZIKV neuropathogenesis.^58^^,^^59^ In Ifnar1^−/−^ mice, ZIKV infection leads to the recruitment of large numbers of CD8^+^ T cells into the CNS, where they help control viral replication in neurons but also contribute to neuroinflammation, neurodegeneration, and paralysis through cytotoxic mechanisms.^58^^,^^59^ This aligns with our observation of CD3^+^CD38^+^ T cell accumulation in the brain during the late stage of infection, suggesting that, beyond their antiviral role, activated T cells may contribute to NAD^+^ depletion and neuronal injury, thereby exacerbating the neurological manifestations of ZIKV infection.

In addition to the lymphoid infiltrate, CD11b^+^TMEM119^-^ macrophages also showed elevated CD38 levels, in line with their increased accumulation during ZIKV infection, suggesting functional activation and a potential role in NAD^+^ consumption. Microglial expansion was also evident, consistent with microgliosis, but these TMEM119^+^ cells did not show increased CD38 expression and are therefore unlikely to contribute directly to the enzymatic depletion of NAD^+^. Altogether, these findings suggest that CD38-mediated NAD^+^ loss in ZIKV-infected brains may result from the combined activity of infiltrating lymphocytes and activated macrophages, rather than from resident microglia.

Although our study was not designed to directly compare ZIKV infection with other viral pathogens, several findings likely reflect general antiviral mechanisms. For instance, transcriptional induction of noncanonical PARP isoforms (e.g., PARP10, PARP12), along with NAD^+^ depletion, has been observed across viral infections, including coronaviruses, where similar activation of MARylating PARPs contributes to NAD^+^ loss.^32^ In contrast, features such as the magnitude and persistence of NAD^+^ depletion in the brain, prolonged infiltration of immune cells expressing CD38, and specifically heightened CD38 activity in astrocytes and infiltrating lymphocytes, may be more characteristic of ZIKV’s neurotropism or unique neuroinflammatory profile. Here, we show that CD38 expression on activated immune cells, including macrophages and lymphocytes, seems to be a hallmark of ZIKV infection. Future comparative studies using other neurotropic viral models will be necessary to delineate which of these effects are specific to ZIKV.

One question that may arise is how CD38, expressed on the surface of immune cells, can promote a decrease in NAD^+^ levels within a tissue, considering that NAD^+^ is an intracellular metabolite. An important aspect to consider is that CD38 also degrades NMN, a key precursor of NAD^+^. In the context of aging, the increased infiltration of CD38^+^ macrophages—recruited by the senescence-associated secretory phenotype (SASP) secreted by senescent cells—into the liver and white adipose tissue has been shown to result in NAD^+^ depletion due to NMN degradation, thereby limiting the availability of NAD^+^ precursors in these tissues.^37^ A similar phenomenon may occur in the brains of ZIKV-infected mice.

Altogether, our findings identify CD38 as a key mediator of NAD^+^ depletion in ZIKV-infected brains, providing mechanistic insights into virus-induced metabolic dysregulation. These results open new perspectives for targeting CD38 as a potential therapeutic approach to mitigate the long-term neurological consequences of ZIKV infection. Future studies should assess whether CD38 inhibition, alone or in combination with NAD^+^ precursor supplementation, can improve neurological outcomes in ZIKV-infected models.

### Limitations of the study

Although we demonstrated that CD38 is responsible for NAD^+^ depletion in the later stages of ZIKV infection in mice, the functional consequences of this event—such as potential neurological or cognitive dysfunction—remain to be determined in future studies. Our data indicate a temporal sequence in which inflammation precedes the infiltration of CD38-expressing immune cells; however, we did not experimentally demonstrate whether preventing neuroinflammation would block this sequence of events. Moreover, although we found a temporal correlation between immune cell infiltration and NAD^+^ decline in the brain, we cannot assert that infiltrating immune cells are directly responsible for the observed NAD^+^ depletion, as no experiment specifically addressed this point. Finally, while our flow cytometry analysis focused on immune cell populations, we did not explore the contribution of resident brain cells, including endothelial cells, which could also play a role in CD38 upregulation and NAD^+^ metabolism. Additional analyses, such as CD38 immunostaining of brain sections, would be valuable to address these aspects.

## Resource availability

### Lead contact

Further information and requests for resources should be directed to the lead contact, Dr. Julianna Dias Zeidler (julianna.zeidler@biof.ufrj.br).

### Materials availability

This study did not generate new unique reagents.

### Data and code availability

• All data required for the main findings of this article are included in the article (main text, figures, and supplemental material).
• This study does not report the original code. Designed primer sequences are available in the key resources table.
• The original datasets or any additional information can be made available from the lead contact upon requests.

## Acknowledgments

This study was supported by the 10.13039/501100004263Fundação de Amparo à Pesquisa do Estado do Rio de Janeiro (FAPERJ E-26/211.318/2021 and E-26/201.173/2021), 10.13039/501100003593Conselho Nacional de Desenvolvimento Científico e Tecnológico - 10.13039/501100003593CNPq, Brazil (312650/2021-3), 10.13039/501100002322Coordenação de Aperfeiçoamento de Pessoal de Nível Superior - 10.13039/501100002322CAPES, Brazil.

## Author contributions

Conceptualization, J.D.Z., A.T.P., and G.N.S.; methodology, G.N.S., J.D.Z., G.F.P, A.T.P., I.N.O.S., and J.C.P.; investigation, G.N.S., J.D.Z., B.G.S., N.M.S.S., L.C.V., R.C.S., T.S.B., M.O.A, L.O.F.S., and I.N.O.S.; writing – original draft, J.D.Z. and G.N.S.; writing – review and editing, J.D.Z., A.T.P., G.N.S., G.F.P., J.C.P., E.N.C.; I.N.O.S., L.O.F.S., M.O.A., T.S.B., R.C.S., L.C.V., N.M.S.S., and B.G.S.; funding acquisition, A.T.P., J.D.Z., G.F.P., and J.C,P. ; resources, E.N.C.; supervision, J.D.Z., A.T.P., and G.F.P.

## Declaration of interests

The authors declare no conflict of interests.

## Declaration of generative AI and AI-assisted technologies in the writing process

During the preparation of this work, the authors used Grammarly and ChatGPT in order to improve grammar and language clarity. After using this tool/service, the authors reviewed and edited the content as needed and take full responsibility for the content of the published article.

## STAR★Methods

### Key resources table

**Table:** 

REAGENT or RESOURCE	SOURCE	IDENTIFIER
**Antibodies**		

CD38 (polyclonal)	R&D Systems	Cat#AF4947
NAMPT Clone: OMNI379	AdipoGen Life Sciences	Cat#AG-20A-0034-C050; RRID: AB_2490118
α-tubulin Clone: B-5-1-2	Sigma-Aldrich	Cat#T5168
HPRT1 Clone: FL-218	Santa Cruz Biotechnology	Cat#sc-20975
CD38-PE Clone: 90/CD38	BD Biosciences	Cat#553764; RRID: AB_395034
CD45-PE-Cy5 Clone: 30-F11	eBioscience	Cat#15-0451-81; RRID: AB_468751
CD11b-FITC Clone: M1/70	eBioscience	Cat#11-0112-82; RRID: AB_464935
CD3-Pacific Blue™ Clone: 17A2	BioLegend	Cat#100213; RRID: AB_493644
CD19-APC Clone: ID3	BD Biosciences	Cat#550992
TMEM119 Clone: V3RT1GOsz	Invitrogen	Cat#14-6119-82; RRID: AB_2848273
Sheep IgG (H + L), HRP-conjugated	Invitrogen	Cat#A16041; RRID: AB_2534715
Mouse IgG (H + L), HRP-conjugated	ThermoFisher Scientific	Cat#31430; RRID: AB_228307
Rat IgG2a Alexa Fluor® 647 Clone: MRG2a-83	BioLegend	Cat#407511; RRID: AB_2716139
Mouse IgG (H + L), IRDye® 680RD-conjugated	LI-COR Biosciences	Cat#926-68070
Mouse IgG (H + L), IRDye® 800CW-conjugated	LI-COR Biosciences	Cat#926-32210
Ab68 anti-CD38	TeneoBio	UniAb clone ID337468

**Bacterial and virus strains**		

Asian ZIKV PE243, Gene bank: KX197192	Isolated from patient (Coelho, 2017)	N/A

**Biological samples**		

Brain tissues	This study	N/A

**Chemicals, peptides, and recombinant proteins**		

Alcohol dehydrogenase (ADH)	Sigma-Aldrich	Cat#A3263
Diaphorase	Sigma-Aldrich	Cat#D5540
Riboflavin 5′-monophosphate sodium salt hydrate	Sigma-Aldrich	Cat#F8399
Resazurin sodium salt	Sigma-Aldrich	Cat#199303
Protease Inhibitor Cocktail	Sigma-Aldrich	Cat#P8340
Nicotinamide 1,N^6^-ethenoadenine dinucleotide (ε-NAD)	Sigma-Aldrich	Cat#2630
78c (CD38 inhibitor)	GSK (Tarrago, 2018)	N/A
Percoll®	Sigma-Aldrich	Cat#P4937

**Critical commercial assays**		

SV Total RNA Isolation System	Promega	Cat#Z3105
High-Capacity cDNA Reverse Transcription Kit	Applied Biosystems	Cat#4368814
SYBR™ Green Master Mix	Applied Biosystems	Cat#4309155
TaqMan® Master Mix	Applied Biosystems	Cat#4304437
Murine IL-6 Standard TMB ELISA Development Kit	PeproTech	Cat#900-T50

**Experimental models: organisms/strains**		

Mouse: Swiss	Charles River Laboratories and bred in our facility	N/A

**Oligonucleotides**		

ZIKV (forward) 5′-CCGCTGCCCAACACAAG-3′	Integrated DNA Technologies	N/A
ZIKV (reverse) 5′-CCACTAACGTTCTTTTGCAGACAT-3′	Integrated DNA Technologies	N/A
ZIKV (probe) 5’-/56-FAM/AGCCTACCT/ZEN/TGACAAGCAATCAGACACTCAA/3IABkFQ/-3′	Integrated DNA Technologies	N/A
*Parp12* (forward) 5′-AGACCGGGAAGAACTGTAGGA-3′	Exxtend Biotecnologia	N/A
*Parp12* (reverse) 5′-TTTGGAAGGAGCAAGAGCCG-3′	Exxtend Biotecnologia	N/A
*Parp10* (forward) 5′-CAAGATCCTGCAGATGCAAA-3′	Exxtend Biotecnologia	N/A
*Parp10* (reverse) 5′-TTGGAGAAGCACACGTTCTG-3′	Exxtend Biotecnologia	N/A
*Parp1* (forward) 5′-GCAGCTTCTGGAGGACGACA-3′	Exxtend Biotecnologia	N/A
*Parp1* (reverse) 5′-TGCCAGGCATTCCCAGTCTT-3′	Exxtend Biotecnologia	N/A
*Sarm1* (forward) 5′-ATTCTGGCACATTGGCGCTT-3′	Exxtend Biotecnologia	N/A
*Sarm1* (reverse) 5′-CGACGAATCGAGCAACAGCA-3′	Exxtend Biotecnologia	N/A
*Cd157* (forward) 5′-AGGAGCCTATCCCACGAGAG-3′	Exxtend Biotecnologia	N/A
*Cd157* (reverse) 5′-CTTCGCCACAGGATTCCACA-3′	Exxtend Biotecnologia	N/A
*Cd38* (forward) 5′-GAAGACTACGCCCCACTTGTT-3′	Exxtend Biotecnologia	N/A
*Cd38* (reverse) 5′-ATTGATGGGCCAGGTGTTTGG-3′	Exxtend Biotecnologia	N/A
*Nampt* (forward) 5′-ACTGTGGCGGGAATTGCTCT-3′	Exxtend Biotecnologia	N/A
*Nampt* (reverse) 5′-TTCCCCCAAGCCGTTATGGT-3′	Exxtend Biotecnologia	N/A
*Il-6* (forward) 5′-CACGGCCTTCCCTACTTCACA-3′	Integrated DNA Technologies	N/A
*Il-6* (reverse) 5′-AGACAGGTCTGTTGGGAGTGGT-3′	Integrated DNA Technologies	N/A
*Tnf* (forward) 5′-TGCTCCAGTGCGGACATCAA-3′	Integrated DNA Technologies	N/A
*Tnf* (reverse) 5′-CGGTGCTGACAGGATGGTCT-3′	Integrated DNA Technologies	N/A
*Ccl-5*/*Rantes* (forward) 5′-CCATATGGCTCGGACACCACT-3′	Integrated DNA Technologies	N/A
*Ccl-5*/*Rantes* (reverse) 5′-TTCTCTGGGTTGGCACACACT-3′	Integrated DNA Technologies	N/A
*Tbp* (forward) 5′-TTCTGCGGTCGCGTCATTTT-3′	Exxtend Biotecnologia	N/A
*Tbp* (reverse) 5′-GGAAGGCTGTTGTTCTGGTC-3′	Exxtend Biotecnologia	N/A

**Software and algorithms**		

SoftMax	Molecular Devices	N/A
QuantStudio™ Design & Analysis	Applied Biosystems	N/A
Image Lab 6.1	Bio-Rad	N/A
BD FACSDiva™	BD Biosciences	N/A
FlowJo™ v10.2	BD Bioscience	N/A
GraphPad Prism 8.0.1	GraphPad/Dotmatics	N/A

### Experimental model and study participant details

Experiments were performed using Swiss mice. Animals were bred and maintained under controlled conditions of temperature (22 ± 2°C) and humidity (50–80%), with a 12:12-hour light–dark cycle, and had free access to food and water. Experiments were conducted using animals from at least three independent litters per experimental condition. Both male and female animals were assigned to the experimental groups. All experimental procedures were approved by the Ethical Committee for Animal Experimentation of the Federal University of Rio de Janeiro (protocols number A11/23-111/21 and 060/25) and were conducted in accordance with the National Institutes of Health Guidelines for the Care and Use of Laboratory Animals.

### Method details

#### *In vivo* experimental procedures

ZIKV infections were performed using an isolate from Pernambuco, Brazil (GenBank accession: KX197192). The neonatal infection model previously established by our group^29^ was employed, in which newborn mice (postnatal day 3, P3) were subcutaneously injected in the dorsal region with 2.7 × 10^5^ plaque-forming units (PFU) of ZIKV or an equivalent volume of mock solution (vehicle) as control. Litters were randomly assigned to the experimental groups (ZIKV-infected or mock-injected), and all pups within each litter received the same treatment to prevent cross-contamination.

For Ab68 treatment, mice were anesthetized with 2.5% isoflurane (Cristália, São Paulo, Brazil) using a vaporizer system (Norwell, MA, USA). An unilateral intracerebroventricular (i.c.v.) injection of the anti-CD38 antibody Ab68 (5.76 μg) was administered freehand using a 2.5 mm-long needle attached to a Hamilton syringe. The injection site was located 1 mm to the right of the midline, equidistant from the eyes and aligned with a line drawn along the anterior base of the eyes, as described previously.^42^ Control animals received an equivalent volume of vehicle (sterile PBS). To ensure that only ZIKV-infected mice in which Ab68 effectively inhibited CD38 activity were included, we applied an exclusion criterion: within the Ab68–ZIKV-infected group, only brain samples displaying NAD^+^ hydrolase activity within the standard deviation range of the mock vehicle control group were included in the analysis.

Except for the flow cytometry analyses (as described in the flow cytometry section), animals were euthanized by cervical dislocation, and brains were dissected at designated time points post-infection, immediately frozen in liquid nitrogen, and stored at −80 °C until further processing.

#### NAD^+^ quantification

NAD^+^ levels were measured using a coupled cyclic enzymatic assay previously described.^59^ Briefly, frozen brains were sectioned coronally and mechanically homogenized in 10% trichloroacetic acid (TCA) using an Ultra-Turrax® homogenizer (IKA-Werke GmbH & Co. KG, Staufen, Germany). Precipitates were resuspended in 0.2 N NaOH for total protein determination, which was used to normalize NAD^+^ concentrations. Supernatants containing soluble metabolites were collected, diluted in phosphate buffer (1 M Na_2_HPO_4_; 1 M NaH_2_PO_4_; pH 8.0), and added to a reaction mixture containing 0.76% ethanol, alcohol dehydrogenase (ADH, 27.2 U/mL; Sigma-Aldrich), diaphorase (1.8 U/mL; Sigma-Aldrich), riboflavin 5′-monophosphate (FMN, 4 μM; Sigma-Aldrich), and resazurin (8 μM; Sigma-Aldrich). Reactions were monitored using a Spectramax® fluorescence microplate reader (Molecular Devices) for 30 minutes, with measurements taken every 30 seconds. NAD^+^ concentrations were determined by interpolation from a standard curve prepared with known concentrations of NAD^+^.

#### RNA extraction and qPCR

RNA was extracted from coronal sections of frozen brains using the SV Total RNA Isolation System (Promega, Wisconsin, USA), following the manufacturer’s instructions. RNA concentration and purity were assessed using a NanoDrop® 2000 spectrophotometer (ThermoFisher Scientific, Massachusetts, USA). Between 1 and 2 μg of total RNA were reverse transcribed into cDNA using the High-Capacity cDNA Reverse Transcription Kit (Applied Biosystems, Massachusetts, USA), also following the manufacturer’s protocol. Quantitative PCR (qPCR) was performed using cDNA samples, gene-specific primers, and SYBR Green Master Mix (Applied Biosystems). Cycle threshold (Ct) values were normalized to the reference gene *Tbp* (TATA-binding protein), and relative gene expression was calculated using the 2^–ΔΔCt^ method.^60^ For quantification of ZIKV genomic RNA, the TaqMan® system (Applied Biosystems) was used. Cq values were converted to PFU equivalents per milligram of tissue by interpolation against a standard curve prepared using 10-fold serial dilutions of a ZIKV viral stock, normalized by the tissue mass used for RNA extraction. All qPCR reactions were run on a QuantStudio™ 3 Real-Time PCR System (Applied Biosystems).

#### NADase activity assay

Coronal sections of frozen brains were mechanically homogenized using an Ultra-Turrax® homogenizer (IKA) in NETN buffer (100 mM NaCl, 1 mM EDTA, 20 mM Tris-HCl, 0.5% NP-40; pH 8.0) supplemented with a protease inhibitor cocktail (Sigma-Aldrich). The resulting lysates were used to assess total NADase activity using a fluorometric assay as previously described.^61^ Briefly, samples were incubated in sucrose buffer (0.25 M sucrose, 40 mM Tris; pH 7.4) containing the fluorescent NAD^+^ analog 1,N^6^-ethenoadenine nicotinamide dinucleotide (ε-NAD) (Sigma-Aldrich). To evaluate CD38-dependent NADase activity, the same protocol was followed with the inclusion of the specific CD38 inhibitor 78c (Sigma-Aldrich).^62^ The fluorescence generated by ε-NAD hydrolysis was monitored using a Spectramax® fluorescence microplate reader (Molecular Devices) over 30 minutes, with measurements taken every 30 seconds.

#### Western-blotting

Brain extracts used for western blotting were prepared as described in the NADase activity section. Proteins were separated by electrophoresis on 15% SDS–polyacrylamide gels (SDS-PAGE), transferred to nitrocellulose membranes (Bio-Rad, California, USA), and probed with primary antibodies against CD38 (AF4947, R&D Systems, Minnesota, USA) or NAMPT (AG-20A-0034-C050, AdipoGen Life Sciences, California, USA), using concentrations recommended by the manufacturers and previously validated in-house. As loading controls, primary antibodies against α-tubulin (T5168, Sigma-Aldrich) and HPRT1 (FL-218, Santa Cruz Biotechnology, Texas, USA) were used. Membranes labeled for CD38 and α-tubulin were developed by chemiluminescence using HRP-conjugated secondary antibodies against sheep (A16041, Invitrogen) and mouse (31430, ThermoFisher Scientific), along with the Pierce™ ECL Western Blotting Substrate (ThermoFisher Scientific), which contains luminol and hydrogen peroxide. Detection was performed using a ChemiDoc™ Imaging System (Bio-Rad). Membranes labeled for NAMPT and HPRT1 were developed using IRDye®-conjugated secondary antibodies against mouse (680RD) and rabbit (800CW) (LI-COR Biosciences), and scanned using the Odyssey® Imaging System (LI-COR Biosciences, Lincoln, USA). Band intensities were quantified using Image Lab 6.1 software (Bio-Rad).

#### Enzyme-linked immunosorbent assay

For cytokine measurement, brain extracts were also prepared as described in the NADase activity section. Brain Il6 concentrations were determined using a commercial kit (PeproTech, New Jersey, USA), according to the manufacturer’s instructions. Absorbance values were obtained in a Spectramax® fluorescence microplate reader (Molecular Devices).

#### Flow cytometry staining and analysis

For analysis of immune and inflammatory cells by flow cytometry, ZIKV-infected and mock-injected mice were anesthetized with an intraperitoneal injection of ketamine (90 mg/kg) and xylazine (4.5 mg/kg) (Sigma-Aldrich). Animals were then subjected to transcardiac perfusion with ice-cold PBS. Brains were immediately dissected and dissociated both enzymatically (collagenase incubation) and mechanically (passage through 40–70 μm cell strainers; Sigma-Aldrich). Immune cells were enriched using a Percoll® (Sigma-Aldrich) density gradient, followed by a serial centrifugation protocol to remove subcellular debris, lipid-rich components, and high-density cell populations. Cell suspensions were pre-incubated on ice with 5% fetal bovine serum (FBS) in PBS to block nonspecific binding, followed by staining with fluorochrome-conjugated antibodies for 15 minutes on ice. Samples were acquired using a FACSCanto II flow cytometer (BD Biosciences), collecting at least 200,000 events per sample, and acquisition was performed using BD FACSDiva™ software (BD Biosciences). Data were analyzed with FlowJo™ v10.2 software. The following antibodies were used: PE rat anti-mouse CD38 (553764, BD Biosciences), PE-Cy5 anti-mouse CD45 (15-0451-81, eBioscience), FITC anti-mouse CD11b (11-0112-82, eBioscience), Pacific Blue anti-mouse CD3 (100213, BioLegend), APC rat anti-mouse CD19 (550992, BD Biosciences), rat anti-mouse TMEM119 (14-6119-82, Invitrogen), Alexa Fluor® 647 anti-rat IgG2a (407511, BioLegend) – used as a secondary antibody for TMEM119 detection.

### Quantification and statistical analysis

Statistical analyses were performed using GraphPad Prism 8.0.1 (GraphPad Software, California, USA). Data are presented as mean ± standard deviation (SD) or standard error of the mean (SEM), as specified in the figure legends. The Kolmogorov–Smirnov test was used to assess the normality of data distribution. Based on the results, either parametric or non-parametric tests were applied to evaluate differences between groups. Comparisons between two groups were conducted using the Student’s t-test. Linear regression analysis was used to assess correlations. Outliers were identified and excluded from all analyses using Grubbs’ test (Extreme Studentized Deviate method), under the assumption of normal distribution. Differences were considered statistically significant when p < 0.05, and defined as ∗p ≤ 0.05; ∗∗p ≤0.01; ∗∗∗p ≤ 0.001; ∗∗∗∗p ≤ 0.0001. Only significant *p*-values are shown in the graphs. Specific description of the number of experimental subjects per group (n) for each set of data can be found in the figure legends.
