# Supplementary material for: CD38 is a key mediator of NAD+ depletion in the brain of ZIKV-infected mice
Source: iScience. 2025 Nov 12;28(12):114018. doi: 10.1016/j.isci.2025.114018 (PMC12682054; doi:10.1016/j.isci.2025.114018)
Supplement: Document S1. Figures S1 and S2 [file mmc1.pdf]

iScience, Volume *28*

## Supplemental information

# CD38 is a key mediator of $NAD^+$ depletion in the brain of ZIKV-infected mice

**Georgia N. Saraiva, Bruna G. Sousa, Nicole M.S. Souza, Louise C. Vitorino, Raquel C. da Silva, Thiago S. Bacelar, Matheus O. Atella, Lorena O. Fernandes-Siqueira, Isis Nem de Oliveira Souza, Eduardo Nunes Chini, Juliana Camacho-Pereira, Giselle F. Passos, Andrea T. Da Poian, and Julianna D. Zeidler**

# Supplemental Figure

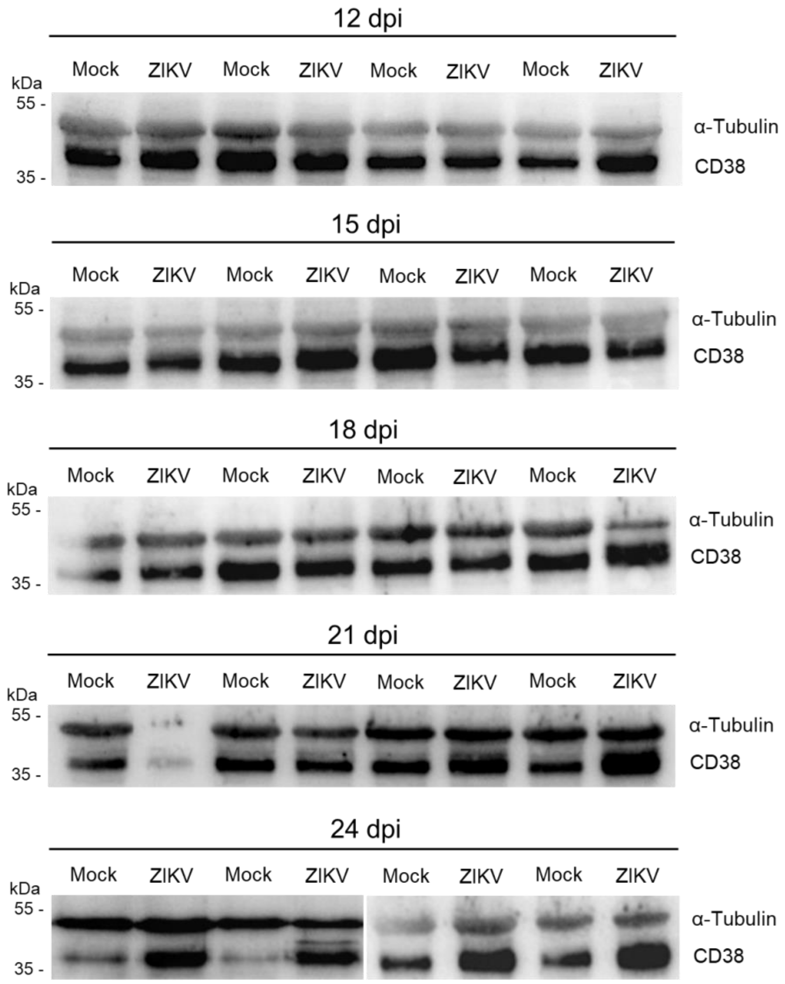


**Figure S1. CD38 is induced in the brain of ZIKV-infected mice.** Western blot of CD38 protein expression in the brains of ZIKV-infected and mock-injected mice at 12-, 15-, 18-, 21-, 24-, and 30-days post-infection (dpi). α-tubulin was used as a loading control (full immunoblot image corresponding to the representative bands shown in Fig. 3L).

Suppl.2

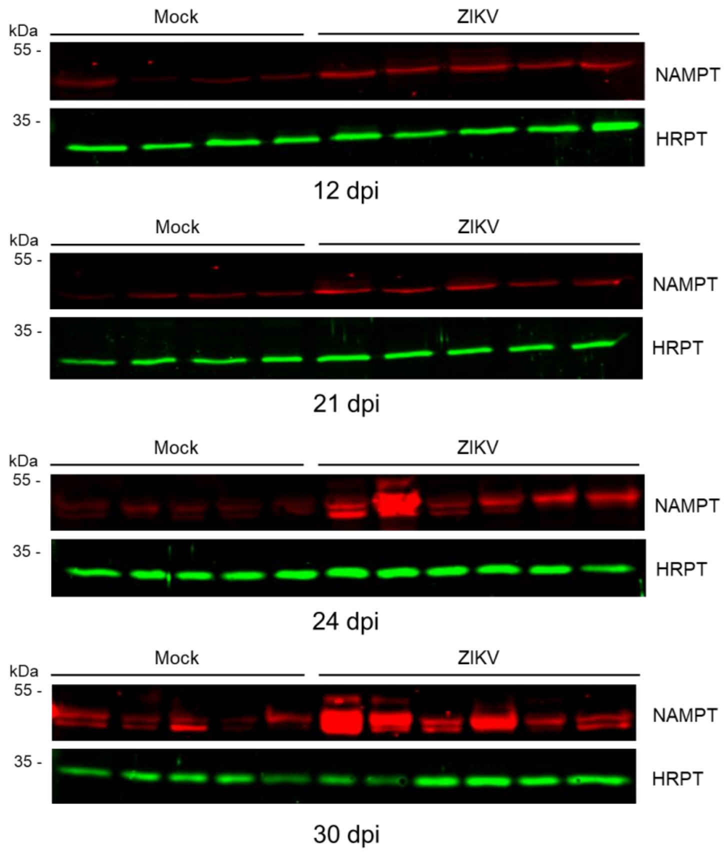


**Figure S2. NAMPT is induced in the brain of ZIKV-infected mice.** Western blot of NAMPT protein expression in the brains of ZIKV-infected and mock-injected mice at 12-, 21-, 24-, and 30-days post-infection (dpi). HPRT was used as a loading control.
